# Retraction Note: Increased Aβ_42_-α7-like nicotinic acetylcholine receptor complex level in lymphocytes is associated with apolipoprotein E4-driven Alzheimer’s disease pathogenesis

**DOI:** 10.1186/s13195-022-01024-5

**Published:** 2022-06-01

**Authors:** Hoau-Yan Wang, Caryn Trocmé-Thibierge, Andres Stucky, Sanket M. Shah, Jessica Kvasic, Amber Khan, Philippe Morain, Isabelle Guignot, Eva Bouguen, Karine Deschet, Maria Pueyo, Elisabeth Mocaer, Pierre-Jean Ousset, Bruno Vellas, Vera Kiyasova

**Affiliations:** 1 Department of Physiology, Pharmacology and Neuroscience, CUNY School of Medicine, 160 Convent Avenue, New York, NY 10031 USA; 2 Department of Biology, Neuroscience Program, Graduate School of The City University of New York, New York, New York 10061 USA; 3 Institut de Recherches Internationales Servier, 50 Rue Carnot, 92284 Suresnes, France; 4 Alzheimer’s Disease Research and Clinical Center, Inserm U1027, Toulouse University Hospital, Toulouse, France; 5 Department of Physiology, Pharmacology & Neuroscience, The City University of New York School of Medicine, CDI-3370 85 St. Nicholas Terrace, New York, NY 10027 USA

**Retraction Note: Alz Res Ther 9, 54 (2017)**

**https://doi.org/10.1186/s13195-017-0280-8**

The Editors-in-Chief have retracted this article. Following publication, concerns have been raised regarding the western blot images presented in Figs. 1, 5 and 6. The authors have provided the raw data, which have been assessed by independent experts and deemed insufficient to address the concerns. The Editors-in-Chief therefore no longer have confidence in the integrity of the data in this article.

Authors Isabelle Guignot, Eva Bouguen, Karine Deschet, Maria Pueyo and Pierre-Jean Ousset agree to this retraction. Author Bruno Vellas agrees to this retraction but disagrees with the Retraction Note. Authors Hoau-Yan Wang, Caryn Trocmé-Thibierge, Elisabeth Mocaer and Vera Kiyasova do not agree to this retraction. Authors Andres Stucky, Sanket M. Shah, Jessica Kvasic and Amber Khan have not responded to any correspondence from the editor or publisher about this retraction. Author Philippe Morain is deceased.

